# Abscisic acid enriched fig extract promotes insulin sensitivity by decreasing systemic inflammation and activating LANCL2 in skeletal muscle

**DOI:** 10.1038/s41598-020-67300-2

**Published:** 2020-06-26

**Authors:** Andrew Leber, Raquel Hontecillas, Nuria Tubau-Juni, Victoria Zoccoli-Rodriguez, Bret Goodpaster, Josep Bassaganya-Riera

**Affiliations:** 1 NIMML Institute, Blacksburg, VA 24060 USA; 2 BioTherapeutics, Blacksburg, VA 24060 USA; 3 AdventHealth Research Institute, Orlando, FL 32804 USA

**Keywords:** Obesity, Type 2 diabetes

## Abstract

Abscisic acid is a phytohormone found in fruits and vegetables and is endogenously produced in mammals. In humans and mice, lanthionine synthetase C-like 2 (LANCL2) has been characterized as the natural receptor for ABA. Herein, we characterize the efficacy of a fig fruit extract of ABA in promoting glycemic control. This ABA-enriched extract, at 0.125 µg ABA/kg body weight, improves glucose tolerance, insulin sensitivity and fasting blood glucose in diet-induced obesity (DIO) and db/db mouse models. In addition to decreasing systemic inflammation and providing glycemic control without increasing insulin, ABA extract modulates the metabolic activity of muscle. ABA increases expression of important glycogen synthase, glucose, fatty acid and mitochondrial metabolism genes and increases direct measures of fatty acid oxidation, glucose oxidation and metabolic flexibility in soleus muscle cells from ABA-treated mice with DIO. Glycolytic and mitochondrial ATP production were increased in ABA-treated human myotubes. Further, ABA synergized with insulin to dramatically increase the rate of glycogen synthesis. The loss of LANCL2 in skeletal muscle abrogated the effect of ABA extract in the DIO model and increased fasting blood glucose levels. This data further supports the clinical development of ABA in the treatment of pre-diabetes, type 2 diabetes and metabolic syndrome.

## Introduction

About 28.3 million Americans have type 2 diabetes (T2D) and over 40.1% of middle-aged adults have pre-diabetes, a disease that has reached pandemic proportions in the U.S. Diabetes is the seventh leading cause of death worldwide, mainly due to its cardiovascular sequelae. Annual expenses exceed $130 billion per year in the U.S. alone, making it one of the most expensive diseases to treat. Prediabetic or high-risk subjects, i.e. those afflicted by excess body weight, insulin resistance, high blood lipids and hypertension, all hallmarks of the metabolic syndrome, are advised to follow dietary and lifestyle guidelines, which have a very low rate of adherence^1^. Combined with a Western diet, low levels of physical activity lead to insulin insensitivity and inability of skeletal muscle to adequately respond to heightened concentrations of insulin in blood. Skeletal muscle comprises between 30–40% of total human body mass, is a primary site for glucose storage as glycogen upon insulin stimulation, and a responsible for a major portion of energy disposal for dietary calories.

Abscisic acid (ABA) is present in fruits and vegetables in varying concentrations. On average the concentration of ABA is 0.29 mg/kg wet weight of vegetable and 0.62 mg/kg of wet weight of fruit. In addition to dietary sources, ABA is an endogenously produced mammalian hormone. Among other sources, ABA release by β-cells is enhanced by GLP-1 *in vitro*, while ABA stimulates glucose-independent GLP-1 release from entero-endocrine cells *in vitro* and oral ABA increases plasma GLP-1 in fasted rats^2^. Plasma ABA concentrations increase after oral glucose load in healthy subjects^2^. ABA increases translocation of the glucose transporter GLUT4 to the plasma membrane and GLUT-4-dependent glucose uptake^3^. The increase of plasma ABA that occurs after an oral glucose load is impaired in patients with T2D and in women with gestational diabetes (GDM)^4^. Normalization of glucose tolerance after childbirth is paralleled by restoration of both the plasma ABA response to oral glucose and normal fasting ABA levels^4^. Further, extracts containing enriched in ABA content improve glycemic and insulinemic indices after oral glucose load^5^.

The mammalian ABA receptor has been characterized to be LANCL2 with molecular modeling predictions^6^, traditional biochemical assays with purified recombinant LANCL2^7^, and cellular functional assays^8^. The signaling pathway downstream of LANCL2 includes a G-protein-mediated activation of adenylate cyclase, cAMP production and activation of PKA^9^. In addition, LANCL2 facilitates phosphorylation of Akt by mTORC2 via direct physical interactions^10^ leading to GLUT4 translocation and glucose uptake^11^. Increased Akt phosphorylation, GLUT4 translocation and glucose transport occur in ABA-treated cells^3^. We discovered that dietary ABA increases insulin sensitivity and suppresses obesity-related inflammation in obese/diabetic db/db mice^12,13^. Dietary ABA also reduces systolic blood pressure and aortic inflammation^14^. ABA is a generally recognized as safe (GRAS) ingredient. In sub-chronic toxicity studies following a 4-week and a 13-week dietary intervention with different concentrations of ABA in rats, no adverse toxicological effects were seen for 90 d at intakes of up to 20,000 ppm (around 1,500 mg/kg body weight per day). Thus, the ABA/LANCL2 axis has emerged as a new promising target for the treatment of dysfunctional glucose homeostasis and inflammation^15^.

In this manuscript, we further characterize the mechanisms underlying the ability of ABA extract to enhance glycemic control. Through use of conditional-knockout mice and human cells *ex vivo*, we define the metabolic effects of ABA on skeletal muscle and the dependence of ABA on the expression of LANCL2 in skeletal muscle for its efficacy in glycemic control. These data demonstrate that ABA, in particular an enriched fig extract, is a potent insulin-sensitizing compound that that modulates systemic inflammation and skeletal muscle metabolism and thereby it shows promise as a clinical candidate for treating pre-diabetes, type 2 diabetes and metabolic syndrome worldwide.

## Results

### ABA-enriched fig extract improves insulin sensitivity and glycemic control

**Figure 1:**
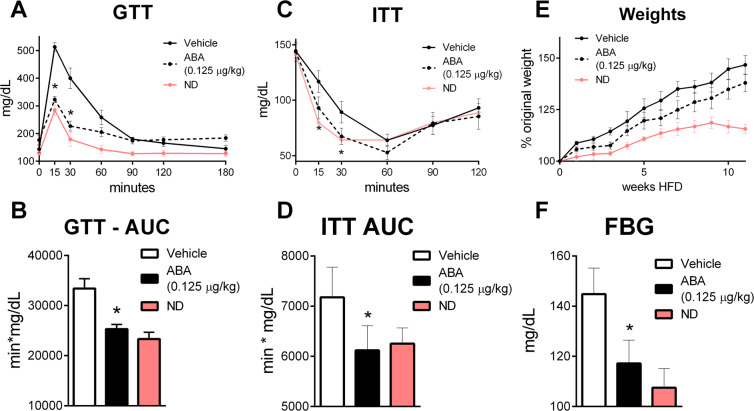
ABA improves glycemic control in a diet-induced model of obesity. Wild-type C57BL/6 mice treated with fig extract ABA (0.125 µg ABA/kg body weight) or vehicle during 12 weeks of 42% kcal from fat diet (TD.88137, Envigo). Intraperitoneal glucose tolerance test (2 g/kg) was conducted at 10 weeks of HFD feeding by serial testing of blood glucose (**A**) and calculation of area under the curve (**B**). Intraperitoneal insulin tolerance test (0.75 U/kg) was conducted at 8 weeks of HFD feeding by serial testing of blood glucose (**C**) and calculation of area under the curve (**D)**. Weekly body weights (**E**) normalized to individual baseline weight. Fasting blood glucose at 12 weeks of HFD feeding (**F**). Wild-type on standard rodent chow were included as controls (ND). (n = 10; *P ≤ 0.05 by treatment).

**Figure 2:**
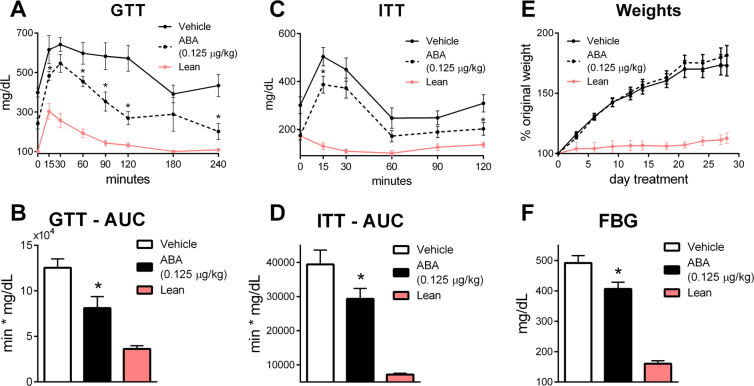
ABA improves glycemic control in a db/db model. Db/db mice were received from Jackson Laboratories, began treatment with fig extract ABA (0.125 µg ABA/kg body weight) at 4 weeks of age and continued on treatment for 4 weeks. Intraperitoneal glucose tolerance test (1 g/kg) was conducted at 3 weeks of treatment by serial testing of blood glucose (**A**) and calculation of area under the curve (**B**). Intraperitoneal insulin tolerance test (0.75 U/kg) was conducted at 2 weeks of treatment by serial testing of blood glucose (**C**) and calculation of area under the curve (**D**). Bi-weekly body weights (**E**) normalized to individual baseline weight. Fasting blood glucose at 4 weeks of treatment (**F**). Lean controls were included (n = 8; *P ≤ 0.05 by treatment).

Using a DIO mouse model of insulin insensitivity, the efficacy of a novel ABA-enriched fig extract in modulation of glycemic control was tested. ABA extract significantly reduced the peak glucose levels after IP glucose load (Fig. 1A), resulting in an overall lower area under the curve in the glucose tolerance test (Fig. 1B). Similarly, mice treated with ABA extract had a greater response to IP insulin resulting in lower blood glucose levels at 15 and 30 in the insulin tolerance test (Fig. 1C) and overall area under the curve (Fig. 1D). ABA extract resulted in a small, non-significant reduction in weight gain over the course of the 12 weeks (Fig. 1E) but normalized fasting blood glucose levels (Fig. 1F). As observed within the DIO model, the ABA extract was also efficacious in a db/db mouse model. In a GTT, ABA-treated mice had a lower peak blood glucose and a faster return to baseline levels (Fig. 2A), resulting in a 40% decrease in area under the curve (Fig. 2B). Following IP insulin, db/db mice receiving ABA extract had lower blood glucose levels throughout the two hours of observation (Fig. 2C), and a significantly lower AUC (Fig. 2D). While no change in weight gain was observed (Fig. 2E), significantly lower fasting blood glucose occurred after four weeks of treatment (Fig. 2F). To benchmark the efficacy of the ABA extract, we compared the 0.125 µg/kg dose of ABA to metformin (100 mg/kg) in the db/db model (Supplemental Fig. 1). Neither ABA extract nor metformin significantly altered weight gain, while both provided a similar response during GTT. In comparison to metformin, ABA extract significantly reduced fasting blood glucose after 5 weeks of treatment (Supplemental Fig. 1).

### ABA extract reduces systemic inflammation during DIO

**Figure 3:**
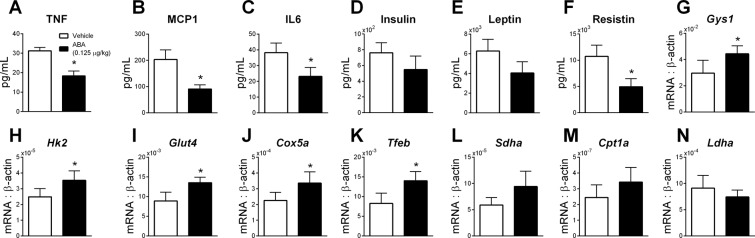
ABA alters systemic inflammatory and metabolic signaling and alters the transcriptional metabolic signatures of skeletal muscle in diet-induced obesity. Wild-type C57BL/6 mice treated with fig extract ABA (0.125 µg ABA/kg body weight) or vehicle during 12 weeks of 42% kcal from fat diet (TD.88137, Envigo). Serum was obtained from whole blood from cardiac puncture post-euthanasia and analyzed for TNF (**A)**, MCP1 (**B**), IL6 (**C**), insulin (**D**), leptin (**E**), and resistin (**F**) by Luminex assay. Gene expression of Gys1 (**G**), Hk2 (**H**), Glut4 (**I**), Cox5a (**J**), Tfeb (**K**), Sdha (**L**), Cpt1a (**M**), and Ldha (**N**) was assessed by qRT-PCR in soleus muscle (n = 10; *P ≤ 0.05 by treatment).

Previously, the therapeutic efficacy of ABA extract in glycemic control has been correlated to lower inflammation in visceral adipose tissue. To assess systemic inflammation, plasma was analyzed for levels of prominent inflammatory cytokines linked to insulin intolerance and obesity-associated inflammation. Treatment with ABA extract resulted in significantly lower levels of TNF (Fig. 3A), MCP-1 (Fig. 3B) and IL-6 (Fig. 3C) in the DIO model. ABA extract resulted in lower TNF-producing macrophages and Th1 cells in visceral adipose tissue in both DIO and db/db models with expansion of regulatory populations, including IL-10-producing macrophages and CD4 + T cells (Treg) (Supplemental Fig. 2). Concentrations of metabolic hormones were also measured in plasma. Insulin (Fig. 3D) and leptin (Fig. 3E) were slightly reduced at a non-significant level, while resistin levels (Fig. 3F) were significantly reduced in the ABA treated group.

### ABA extract increases metabolism in skeletal muscle

**Figure 4:**
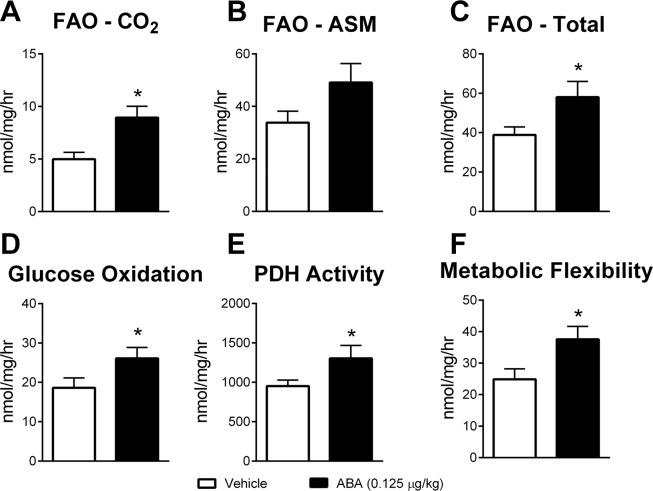
Oral ABA increases metabolic activity in skeletal muscle. Soleus muscle was collected from the hind limbs of mice treated with fig extract ABA (0.125 µg ABA/kg body weight) or vehicle during 12 weeks of 42% kcal from fat diet (TD.88137, Envigo). Complete (**A**), incomplete (**B**), and total (**C**) fatty acid oxidation rates as measured by labelled CO_2_ and acid soluble metabolites after incubation with [1-^14^C] palmitate. Glucose oxidation (**D**), PDH activity (**E**) and metabolic flexibility (**F)** as measured by ^14^CO_2_ production following D[U-^14^C] glucose and non-labelled glucose pre-incubation (n = 10; *P ≤ 0.05 by treatment).

With decreased insulin levels and only mild effects on weight gain, we hypothesized that the efficacy of ABA-enriched fig extract was tied to changes in metabolic activity of skeletal muscle. In accordance with this hypothesis, we assessed gene expression of critical metabolic markers in soleus muscle of DIO mice treated with ABA extract. Significant increases in glycogen synthase (Gys1, Fig. 3G), hexokinase 2 (Hk2, Fig. 3H), glucose transporter 4 (Glut4, Fig. 3I) and cytochrome c oxidase subunit 5a (Cox5a, Fig. 3J) were observed in addition to significantly higher expression of transcription factor EB (Tfeb, Fig. 3K). In parallel to these significant changes, slight increases in succinate dehydrogenase (Sdha, Fig. 3L) and carnitine palmitoyltransferase Ia (Cpt1a, Fig. 3M) were also observed with slight, non-significant reduction of lactate dehydrogenase expression (Ldha, Fig. 3N). To examine these metabolic changes at a functional level, cells from the soleus muscle of DIO mice treated with ABA extract were isolated. In studies with labeled palmitate, cells from ABA-treated mice displayed significantly greater complete and total fatty acid oxidation, with slightly increased incomplete fatty acid oxidation (Fig. 4A–C). In studies with labeled glucose, cells from ABA-treated mice displayed significantly increased glucose oxidation (Fig. 4D), PDH activity (Fig. 4E) and metabolic flexibility (Fig. 4F) in comparison to vehicle treated controls.

### Loss of LANCL2 in skeletal muscle diminishes response to ABA-enriched fig extract

**Figure 5:**
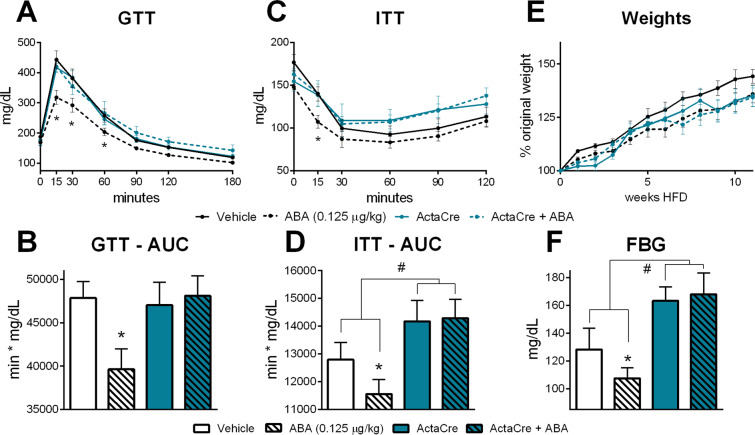
Genetic deletion of LANCL2 in skeletal muscle abrogates effects of ABA on glycemic control. Skeletal muscle-specific specific deletion of LANCL2 was attained by expression of cre-recombinase under control of the Acta1 promoter in Lancl2fl/fl mice. Age-, gender- and litter-matched Lancl2fl/fl;cre- and Lancl2fl/fl;Acta1cre+ mice were placed on 42% kcal from fat diet (TD.88137, Envigo) for 12 weeks. Oral fig extract ABA (0.125 µg ABA/kg body weight) was administered daily. Intraperitoneal glucose tolerance test (2 g/kg) was conducted at 10 weeks of HFD feeding by serial testing of blood glucose (**A**) and calculation of area under the curve (**B**). Intraperitoneal insulin tolerance test (0.75 U/kg) was conducted at 8 weeks of HFD feeding by serial testing of blood glucose (**C**) and calculation of area under the curve (**D**). Weekly body weights (**E**) normalized to individual baseline weight. Fasting blood glucose at 12 weeks of HFD feeding (F) (n = 10; *P ≤ 0.05 by treatment; ^#^*P* ≤ 0.05 by genotype).

After observation of the effects of ABA on skeletal muscle, we generated muscle-specific knockouts of LANCL2, the mammalian ABA receptor, to determine the contribution of skeletal muscle LANCL2 signaling to the overall glycemic response to ABA-enriched fig extract. The loss of LANCL2 in skeletal muscle (ActaCre) abrogated the effect of ABA extract during GTT in mice (Fig. 5A,B). Similarly, no efficacy of ABA extract was observed in an ITT in ActaCre mice (Fig. 5C,D) with significantly higher AUC in both ABA-treated and untreated ActaCre mice (Fig. 5D) due to an accelerated return to baseline in these groups. Altered weight gain was not observed in ActaCre mice (Fig. 5E). ABA extract did not affect fasting blood glucose in ActaCre mice, with both untreated and ABA-treated groups displaying higher blood glucose concentrations than Lancl2fl/fl;cre- controls (Fig. 5F). Beyond glycemic control, ActaCre mice also had diminished responses to ABA extract in terms of immunological changes in visceral adipose tissue (Supplemental Fig. 2). Effects on macrophages were abrogated, while CD4 + T cell effects were greatly reduced.

### ABA increases insulin-independent metabolism in human skeletal muscle

**Figure 6:**
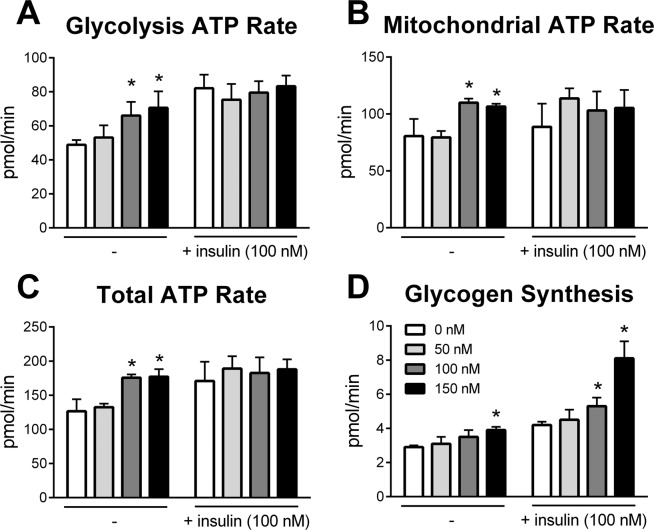
ABA increases the metabolic activity of human skeletal myotubes *ex vivo*. Human skeletal myoblasts were differentiated into myotubes for 72 h. Cell were treated with ABA (0, 50, 100, 150 nM) and human insulin lispro (0, 100 nM) 1 h prior to assay. Glycolysis ATP rate (**A**), mitochondrial ATP rate (**B**), and total ATP rate (**C**) were measured by Seahorse XF Real-Time ATP Rate Assay. Glycogen synthesis rate was measured by solubilization of cells after 4 h incubation with ABA, labeled glucose and insulin (**D**) (n = 9; *P ≤ 0.05 relative to 0 nM).

To determine if ABA had similar effects in human skeletal muscle, we cultured differentiated myotubes with ABA (0, 50, 100, 150 nM) in the presence and absence of insulin (100 nM). As observed with mice, treatment of human muscle cells with ABA resulted in increased metabolic activity with significantly higher glycolytic (Fig. 6A), mitochondrial (Fig. 6B) and total ATP (Fig. 6C) production rates at concentrations of 100 and 150 nM. Notably, ABA alone provided similar increases to glycolytic and total ATP production when compared to insulin alone; although little to no synergistic effect was observed in ATP production. In contrast, ABA alone provided moderate increase to the rate of glycogen synthesis, but nearly doubled the rate in combination with insulin (Fig. 6D). When myoblasts were differentiated in the presence of inflammatory stimuli (palmitate, TNF), ABA was observed to lessen inflammatory responses and increase markers of insulin sensitivity (Supplemental Fig. 5). With palmitate stimulation, ABA lessens activity of NF-κB p65 and expression of IL-6 while increasing Pgc1a expression in the presence and absence of insulin and GYS1 expression in the presence of insulin. With TNF stimulation, ABA with insulin provided a numerical increase in glycogen production over insulin alone and a statistical increase over vehicle control. Additionally, ABA and insulin had statistically higher CKM expression relative to insulin alone.

## Discussion

Skeletal muscle is responsible for between 50–90% of glucose uptake in humans^16^. Mechanisms linking skeletal muscle to insulin resistance are numerous. Impaired mitochondrial metabolism leads to increased intramyocellular fat content and inflammation^17,18^. Signal transduction between IRS-1 and PI3K and other kinases is impaired leading to lower glucose uptake and other effects^19^. Free fatty acid turnover inhibits PDH activity and lowers glucose oxidation^20^. Decreased glycogen storage contributes to increased fatty acid uptake and accumulation of lipid intermediates, which impair insulin signaling^21^. These mechanisms intertwine and overlap making type 2 diabetes a complex disease that remains difficult to manage. The data presented in this manuscript and the published literature on ABA to date, highlight the ability of this compound to intersect the pathogenesis of type 2 diabetes and insulin resistance at multiple levels. ABA treatment, both at the organism levels and in specific muscle cells *ex vivo*, increases both glucose and fatty acid metabolism in the mitochondria, increases glycogen synthesis, activates PI3K independently of insulin and promotes GLUT4 translocation to the cell membrane.

Endogenous production of ABA has been previously linked to hormonal regulation *in vitro*. Specifically, GLP-1 increases ABA release from β cells, while ABA has been shown to increase insulin secretion from pancreatic islets and cell lines^22^. Compelling *in vitro*, the *in vivo* data, presented herein, supports the therapeutic efficacy of ABA independent of these mechanisms for glycemic control. Fig extract ABA increases glucose tolerance in IP GTTs. As GLP-1 is primarily released by entero-endocrine cells after oral glucose load, the efficacy of ABA in the context of IP glucose challenge, suggests an ability to function without relying on GLP-1 as a mediator and the potential to synergize with currently available GLP-1 agonists. Further, administration of ABA did not increase plasma insulin levels. In contrast, ABA supplementation provided a small decrease in plasma, suggesting an ability to provide glycemic control in the absence of hyperinsulinemia at doses of 0.125 µg/kg. This indicates increased insulin sensitivity or an ability to support insulin-independent glucose disposal. Either mechanism would serve to alleviate pancreatic stress in type 2 diabetes, during which insulin production is often increased due to diminished responsiveness of cells to insulin stimulus.

Based on previous connections to GLUT4 translocation and the described effects on glycemic control, it is unsurprising that ABA increases the expression of key elements of glucose metabolism, such as *Glut4*, *Hk2*, *Sdha*, and *Gys1*, in skeletal muscle. Linked to these effects and overall muscle metabolism, is the transcription factor, TFEB. More specifically, TFEB activity in skeletal muscle is increased during periods of exercise, by Pgc1a, and by increase in the intracellular calcium levels^23,24^. Importantly, TFEB is a main controller of mitochondrial biogenesis and mitophagy, ensuring sufficient mitochondria are present and in good health relative to energetic demands^25^. In line with this function, we observe increases in important mitochondrial enzymes, *Cox5a* and *Sdha*, as well as the main controller of fatty acid oxidation, *Cpt1a*. As such, ABA treatment likely results in greater mitochondrial mass and enhanced ability of muscle to utilize fatty acids during a resting state. In addition to the increased expression of *Tfeb*, ABA induces many similar effects to those observed in insulin-independent, exercise-induced glucose uptake by muscle, as well as the mechanisms in which exercise restores insulin sensitivity^26^, including GLUT4 translocation, activation of AMPK, and induction of calcium release.

Insulin-stimulated glycogen synthesis is decreased in individuals with type 2 diabetes and metabolic syndrome, accounting for most of the differential in whole-body glucose disposal^16,27^. Activated primarily by PP1 via insulin-stimulation of ISPK1, glycogen synthase is negatively regulated by GSK3. As LANCL2 activation by ABA, leads to insulin-independent upregulation of PI3K/Akt activity, it is likely that this pathway is responsible for the increased glycogen synthesis in human myotubes *ex vivo* that is further enhanced by the presence of insulin. Alternatively, LANCL2 activation may influence the expression of PP1 regulatory subunits known to bind glycogen leading to downstream regulation of PP1 activity. Notably, significant differences in the distribution of glycogen reserves exist between mice and humans, with a much greater proportion of glycogen stored in muscle in humans and an approximate 10-fold increase in glycogen to muscle mass ratio in humans compared to mice^28^. The liver remains an important source of glycogen, particularly to avoid hypoglycemia during fasting. In ActaCre mice, an accelerated increase in blood glucose levels after insulin-induced drop could suggest altered liver glycogen storage, potentially as a compensatory mechanism for impaired glucose disposal and glycogen storage in muscle. However, further work on the interplay of muscle and liver glycogen synthesis in the context of ABA treatment is needed.

Beyond direct effects on skeletal muscle, ABA may further support the maintenance of insulin tolerance in muscle through the muscle/adipose axis. The expression profiles of adipocytes are strongly correlated to the function of muscle cells through secreted products, such as IL-6^29^. Perturbed muscle substrate oxidation in obese and diabetic individuals is a contributing factor to impaired glucose homeostasis and reduced insulin sensitivity^30,31^, and release of inflammatory factors from adipocytes rapidly induce insulin resistance in skeletal muscle using co-culture^32^. Importantly, exposure to inflammatory cytokines, such as TNF and IL-6, and activation of NF-κB in muscle can decrease oxidative metabolism, induce atrophy, and prevent insulin-stimulated Akt phosphorylation, all of which are factors in the development of type 2 diabetes and metabolic syndrome. Additionally, ABA reduced plasma levels of leptin and resistin, two notable adipokines tied to the diminished uptake of glucose in diabetes and impaired IRS-1 signaling^33,34^. Therefore, an ability to influence both direct metabolic pathways in skeletal muscle and the underlying inflammation provides a robust platform for the clinical development of ABA.

ABA is a potent insulin-sensitizing compound with the ability to control systemic glycemic responses and skeletal muscle metabolism at oral doses as low as 0.125 µg/kg. In this manuscript, we provide extensive evidence for the efficacy of an ABA-enriched fig extract in DIO and db/db mouse models of insulin insensitivity. Treatment with standardized ABA extract induced greater insulin sensitivity, decreased fasting blood glucose and improved response to oral glucose load. Thus, ABA in combination with diet and exercise may serve as a frontline intervention in early pre-diabetes, as well as type 2 diabetes.

## Methods

### Mice

Age-, gender- and litter-matched mice were used in all projects. Wild-type, Lancl2fl/fl, and Lancl2fl/fl;Acta1Cre+ mice were bred in-house, maintained in BTI facilities, and placed into projects at 8–10 weeks of age. Db/db mice were obtained from Jackson Laboratories and used beginning at 4 weeks of age. All mice were used in accordance with BioTherapeutics Inc Institutional Animal Care and Use Committee approved protocols. A 42% kcal from fat diet (TD.88137, Envigo) was used for diet-induced obesity (DIO) studies for 12 weeks. Db/db mice were placed on standard rodent chow. Mice were randomized into treatment groups based on baseline fasting blood glucose and weight to provide equal starting point. ABA, in the form of a liquid-enriched fig extract, was provided to mice orally at a dosage of 0.125 µg ABA per kilogram of body weight on a daily basis. Mice were weighed on a daily (db/db) to weekly basis (DIO). Fasting blood glucoses were obtained by tail vein bleeding after a 6-hour fast with an Accu-Chek Aviva glucometer.

### Extract preparation

Extracts were obtained from Euromed (Spain). Pharmaceutical-grade liquid fig fruit extracts were produced from *Ficus carica* L. fruit using a sophisticated, patent-pending process (EP17382616.5) and were standardized in ABA content, as described previously^5^. ABA content in these extracts was determined using reversed-phase ultra-high-performance liquid chromatography (UHPLC).

### Glucose and insulin tolerance tests

Mice were fasted for 4 hours. Glucose (1–2 mg/kg) and insulin (0.75 U/kg) were injected intraperitoneally (IP) after obtaining a baseline fasting blood glucose. Blood glucose levels were assessed at described intervals up to 240 minutes after the glucose challenge or up to 120 minutes after insulin challenge with an Accu-Chek Aviva glucometer. Data was interpreted by time course and by calculated area under the curve for the described timepoints.

### Serum testing

After euthanasia, whole blood was obtained by cardiac puncture and passed through a heparinized tube to prevent clotting. Whole blood was centrifuged and plasma was collected. Cytokines and metabolic hormones were measured using a MAP Luminex assay (Milliplex, MMHMAG-44K) according to manufacturer’s instructions.

### Gene expression

Thirty milligrams of muscle were dissected and weighed from the soleus. RNA was extracted using a Qiagen RNeasy Mini kit. Complementary DNA was synthesized by iScript reverse transcriptase. Data was acquired with SybrGreen reaction mix on a qRT-PCR. Gene expression was quantified by standard curve calculation followed by normalization to expression of beta-actin.

### Human skeletal myoblasts

Primary skeletal myoblasts were obtained from third-party vendor (Lonza), subject consent was obtained by this vendor. Isolated human skeletal myoblasts were plated into 96-well flat-bottom plates, pre-incubated with 10% Matrigel in DMEM. Myoblasts were differentiated by 72 h incubation with DMEM supplemented with 2% horse serum and 1% penicillin/streptomycin. Media was renewed at 48 h. Prior to assay, media was replaced with fresh media containing ABA and/or insulin in the desired concentration. For studies of inflammatory stress, myoblasts were stimulated with palmitate (0.5 mM) of TNF (100 ng/mL) during hours 24–72 of differentiation. For the final 6 h, ABA (100 nM) and insulin (100 nM) were added to the media. For measurement of NF-κB activity, myotubes were lysed and whole cell protein extract was obtained. Quantification of NF-κB was conducted by TransAM NF-κB p65 ELISA assay (ActiveMotif).

### Metabolic assay

For murine samples, cells were isolated from soleus muscle by gentle mechanical dissociation by tissue disruptor. For fatty acid oxidation, cells were pre-incubated for 3 h with [1-^14^C] palmitate (1 mCi/ml; PerkinElmer, Boston, MA) and respective non-labeled palmitate (100 mM). Palmitate was coupled to a fatty acid free BSA in a molar ratio of 5:1. Following incubation, ^14^CO_2_ and ^14^ASM (acid soluble metabolites) were measured^35,36^. For glucose oxidation and metabolic flexibility, cells were pre-incubated with a glucose- and serum-free media for 90 minutes, followed by 3 h incubation with D[U-^14^C] glucose (1 mCi/ml; PerkinElmer, Boston, MA) and 5.5 mM of non-labeled glucose. After ^14^C glucose incubation, ^14^CO_2_ was measured as described^35,36^. Glycogen synthesis was measured as described^35,37^. Glycolysis ATP rate, mitochondrial ATP rate and total ATP rate were determined using a Seahorse XF Real-Time ATP Rate Assay, according to manufacturer’s instructions.

### Statistical analysis

Data are expressed as mean and SEM. Parametric data were analyzed using ANOVA, followed by the Scheffe multiple-comparisons test. ANOVA was performed using the general linear model procedure of SAS (SAS Institute, Cary, NC). Statistical significance was determined at *P* ≤ 0.05.

### Ethics statement

All mice were used in accordance with BioTherapeutics Inc Institutional Animal Care and Use Committee approved protocol (Protocol #19-002-BTI). Human studies were used in accordance with BioTherapeutics Inc Institutional Review Board approved protocol (Protocol #18-003). All experimental protocols were approved by the indicated review committee above.

## Supplemenatry information

Supplemenatry information.
