# Supplementary material for: Abscisic acid enriched fig extract promotes insulin sensitivity by decreasing systemic inflammation and activating LANCL2 in skeletal muscle
Source: Sci Rep. 2020 Jun 26;10:10463. doi: 10.1038/s41598-020-67300-2 (PMC7319979; doi:10.1038/s41598-020-67300-2)
Supplement: Supplementary file 1 — Supplemenatry information. [file 41598_2020_67300_MOESM1_ESM.docx]

**Abscisic acid enriched fig extract promotes insulin sensitivity by decreasing systemic inflammation and activating LANCL2 in skeletal muscle.**

Andrew Leber^1,2^, Raquel Hontecillas^1,2^, Nuria Tubau-Juni^1,2^, Victoria Zoccoli-Rodriguez^1,2^, Bret Goodpaster^3^, and Josep Bassaganya-Riera^1,2^

^1^ NIMML Institute, Blacksburg, VA 24060, USA

^2^ BioTherapeutics, Blacksburg, VA, 24060, USA

^3^ AdventHealth Research Institute, Orlando, FL, 32804, USA


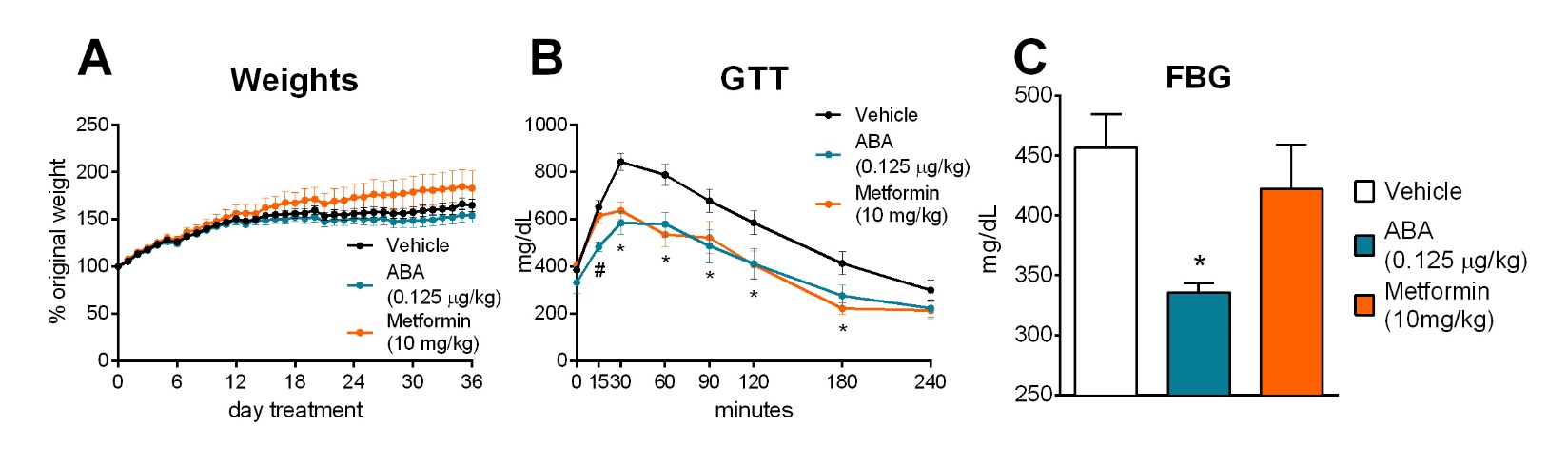


Supplemental Figure 1. Comparative efficacy of oral ABA extract to metformin in a db/db model. Weights of male and female db/db mice treated with fig extract ABA (0.125 µg ABA/kg body weight) or metformin (10 mg/kg) by daily gavage for five weeks (A). Intraperitoneal glucose tolerance test after 30 days of treatment (B) and fasting blood glucose after 35 days of treatment (C). Db/db mice were received from Jackson Laboratories and began treatment at 4 weeks of age (n = 8, **P* ≤ 0.05 relative to vehicle, # *P* ≤ 0.05 relative to vehicle and metformin).


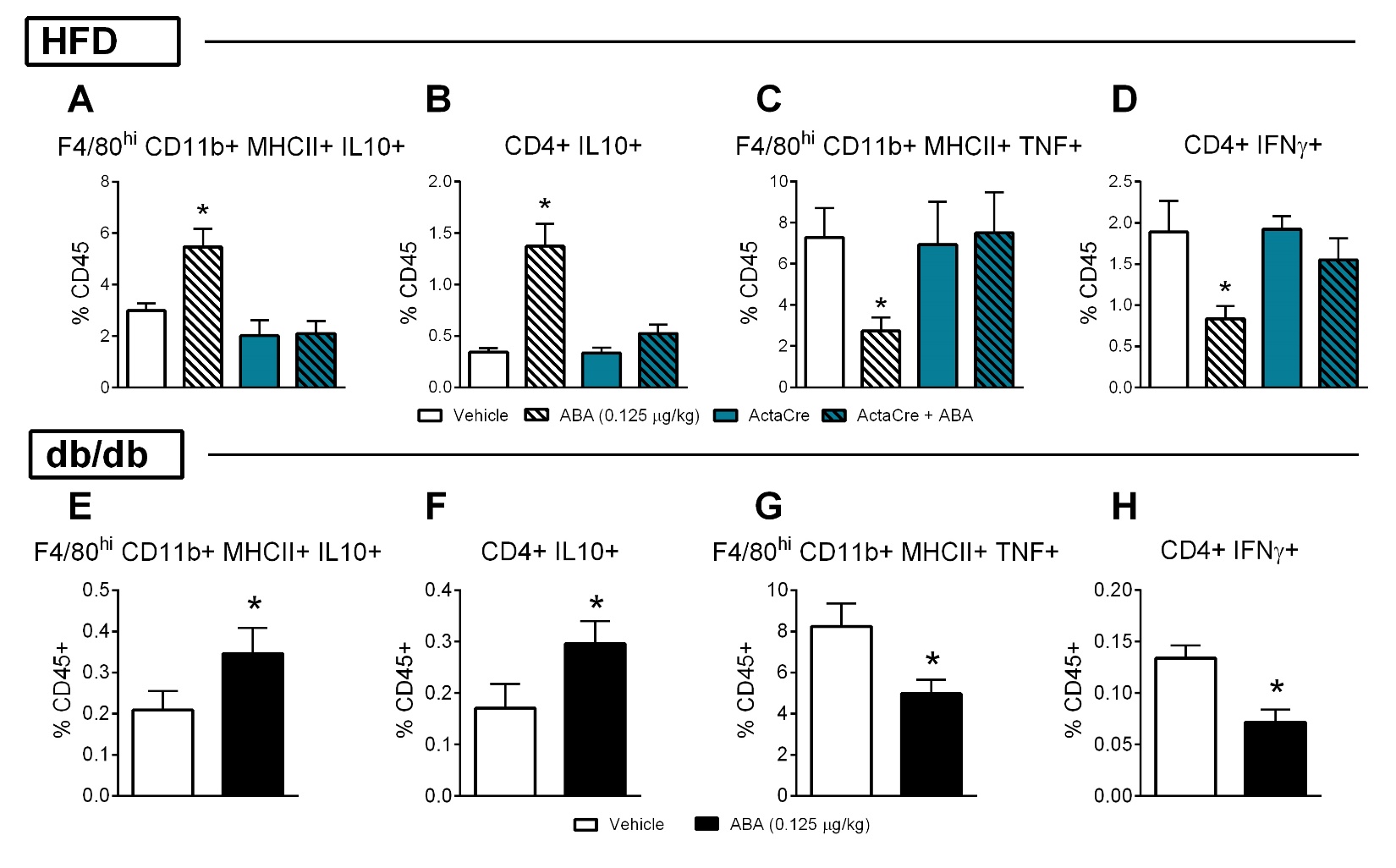


Supplemental Figure 2. ABA decreases white adipose tissue inflammation in diet-induced obesity and db/db models. Visceral white adipose tissue was collected from male and female mice on 42% kcal from fat diet (TD.88137, Envigo) for 12 weeks (A-D) or db/db mice after 4 weeks of treatment (E-H). Adipose tissue was digested and stromal vascular fraction was collected. Cells were stained and analyzed by flow cytometry for F4/80^hi^ CD11b+ MHCII+ IL10+ regulatory macrophages (A, E), CD4+ IL10+ regulatory T cells (B, F), F4/80^hi^ CD11b+ MHCII+ TNF+ inflammatory macrophages (C, G) and CD4+ IFNγ+ Th1 cells (D, H). (HFD, n = 10; db/db n = 8; **P* ≤ 0.05 relative to vehicle).


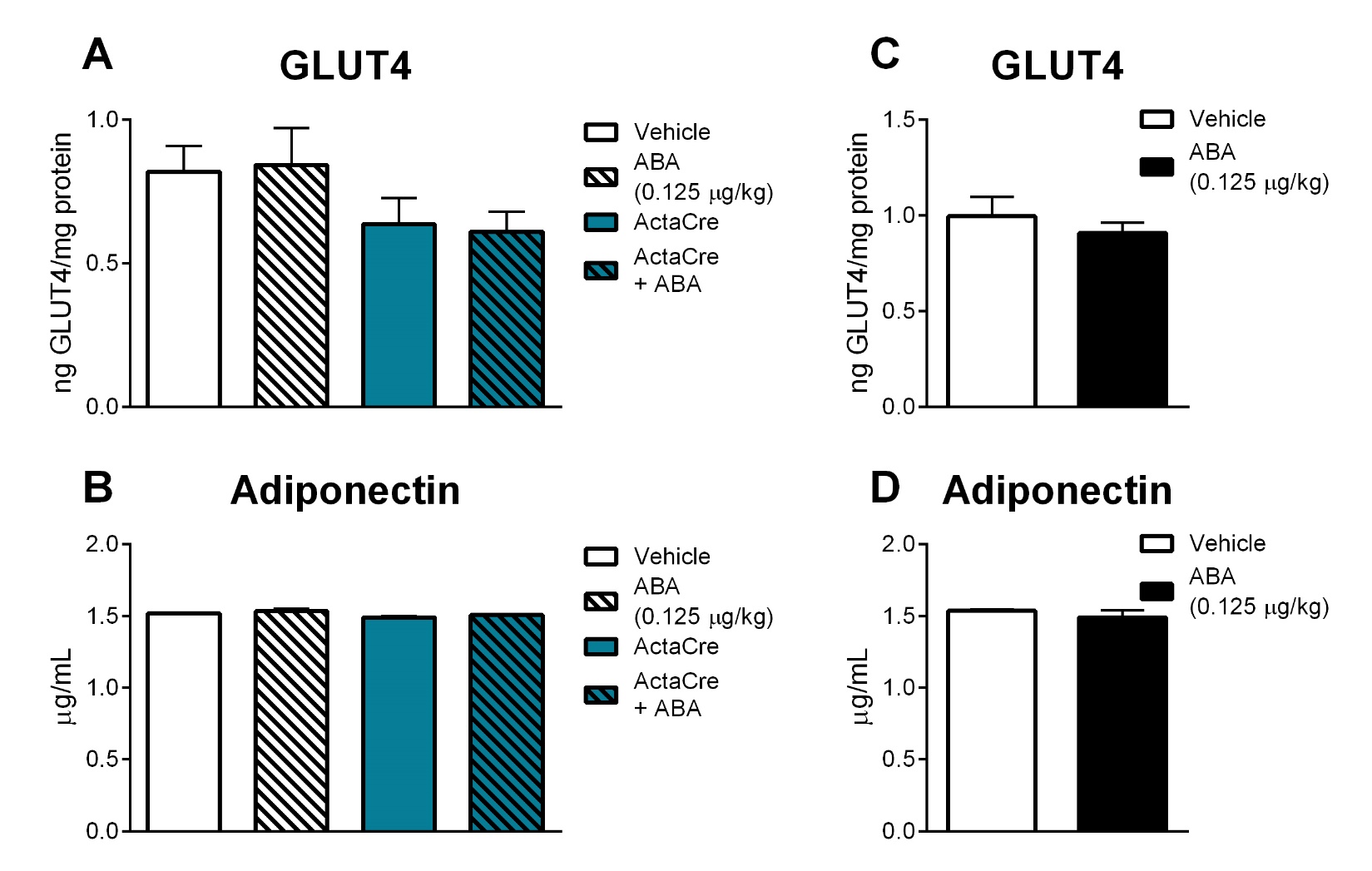


Supplemental Figure 3. Effects of ABA on GLUT4 in skeletal muscle and serum adiponectin in diet induced obesity and db/db models. Skeletal muscle (gastrocnemius) and blood was collected from male and female mice on 42% kcal from fat diet (TD.88137, Envigo) for 12 weeks (A-B) or db/db mice after 4 weeks of treatment (C-D). Protein from skeletal muscle was extracted by cellular lysis and homogenization. GLUT4 expression was measured by ELISA. Adiponectin was measured in serum by ELISA. (HFD, n = 10; db/db n = 8; **P* ≤ 0.05 relative to vehicle).


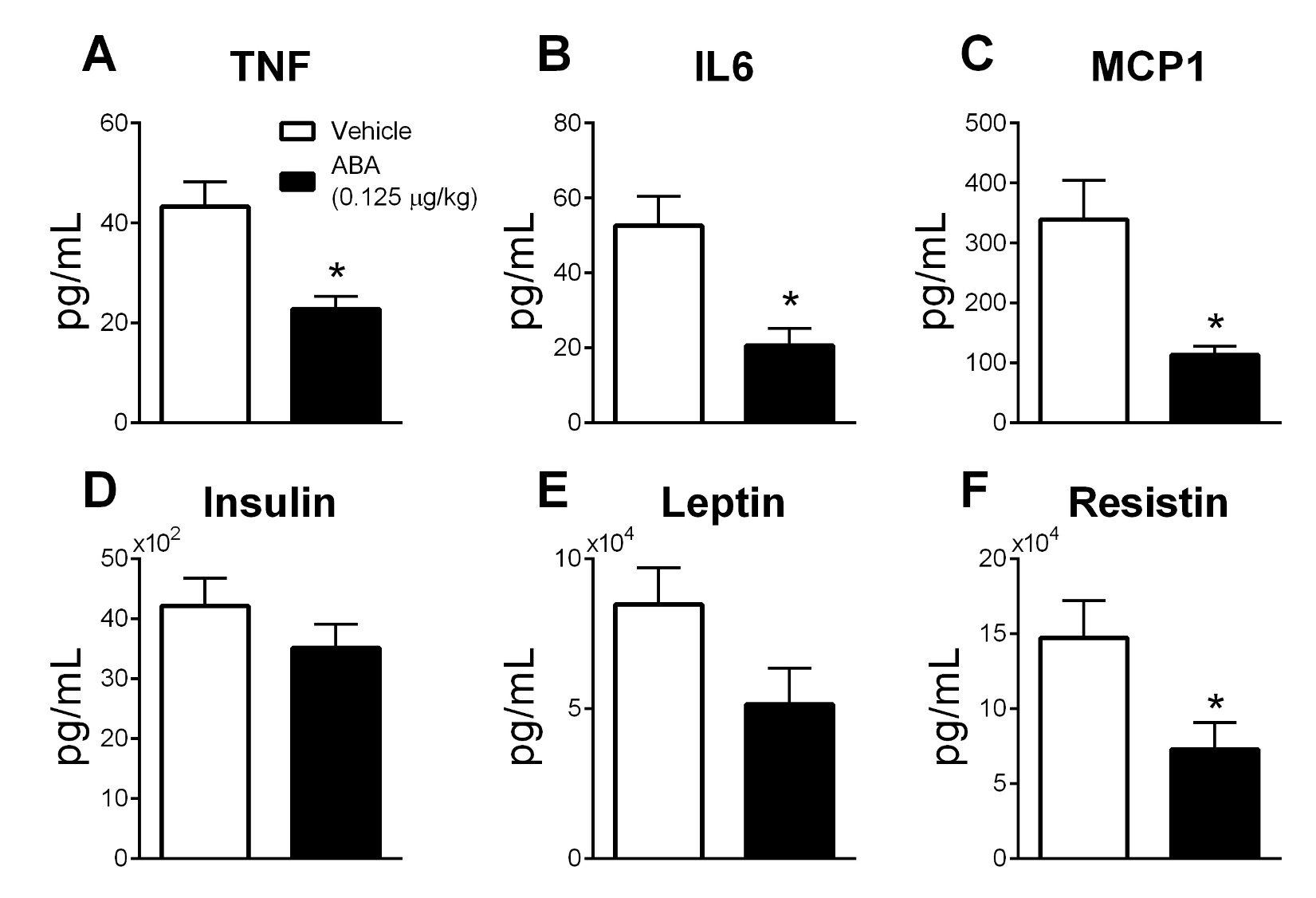


Supplemental Figure 4. ABA alters systemic inflammatory and metabolic signaling db/db mice. Db/db mice were received from Jackson Laboratories, began treatment with fig extract ABA (0.125 µg ABA/kg body weight) at 4 weeks of age and continued on treatment for 4 weeks. Serum was obtained from whole blood from cardiac puncture post-euthanasia and analyzed for TNF (A), IL6 (B), MCP1 (C), insulin (D), leptin (E), and resistin (F) by Luminex assay. (n = 8; **P* ≤ 0.05 relative to vehicle).


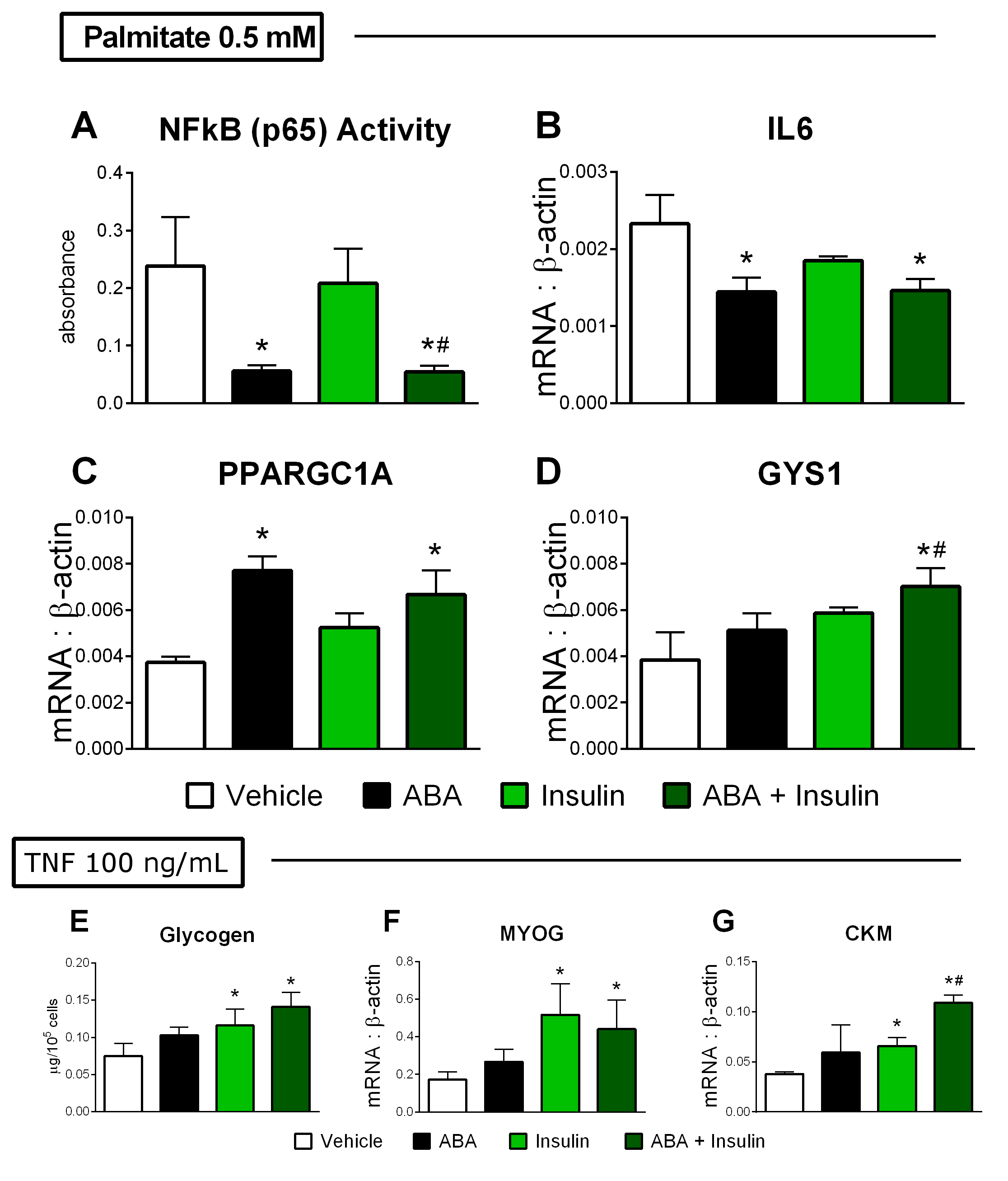


Supplemental Figure 5. ABA alters responses of skeletal muscle to inflammatory stress *ex vivo*. Myoblasts were stimulated with palmitate ([0.5 mM], A-D) or TNF ([100 ng/mL], E-G) during differentiation. Cells were treated with ABA (100 nM), insulin (100 nM) or ABA and insulin together for 6 h prior to assay. Cells were lysed for the collection of protein (NF-κB), glycogen content, or isolation of mRNA. In palmitate stimulated cells, NF-κB activity (A) was measured by ActiveMotif TransAM assay. IL-6 (B), PPARGC1A (C) and GYS1 (D) mRNA expression was evaluated by qRT-PCR. In TNF stimulated cells, glycogen was measured by colorimetric assay (E). MYOG (F) and CKM (G) were evaluated by qRT-PCR. (n = 6; **P* ≤ 0.05 relative to vehicle; ; #*P* ≤ 0.05 relative to insulin).
